# Informing Equitable Water and Food Policies through Accurate Spatial Information on Irrigated Areas in Smallholder Farming Systems

**DOI:** 10.3390/w13243627

**Published:** 2021-12-16

**Authors:** James Magidi, Barbara van Koppen, Luxon Nhamo, Sylvester Mpandeli, Rob Slotow, Tafadzwanashe Mabhaudhi

**Affiliations:** 1 Geomatics Department, Tshwane University of Technology, Pretoria 0001, South Africa; 2 International Water Management Institute (IWMI), Southern Africa Office, Pretoria 0184, South Africa; 3 Water Research Commission of South Africa (WRC), Pretoria 0081, South Africa; 4 Faculty of Science, Engineering and Agriculture, University of Venda, Thohoyandou 0950, South Africa; 5 Centre for Transformative Agricultural and Food Systems (CTAFS), School of Life Sciences, University of KwaZulu-Natal, Scottsville, Pietermaritzburg 3209, South Africa; 6 Department of Genetics, School of Genetics, Evolution & Environment, University College, London WC1E 6BT, UK; 7 Centre for Transformative Agricultural and Food Systems (CTAFS), School of Agricultural, Earth and Environmental Sciences, University of KwaZulu-Natal, Scottsville, Pietermaritzburg 3209, South Africa; 8 International Water Management Institute (IWMI-GH), West Africa Office, Accra GA015, Ghana

**Keywords:** irrigated areas, vegetation indices, informal irrigation, water security, sustainable development smallholder farmers, South Africa

## Abstract

Accurate information on irrigated areas’ spatial distribution and extent are crucial in enhancing agricultural water productivity, water resources management, and formulating strategic policies that enhance water and food security and ecologically sustainable development. However, data are typically limited for smallholder irrigated areas, which is key to achieving social equity and equal distribution of financial resources. This study addressed this gap by delineating disaggregated smallholder and commercial irrigated areas through the random forest algorithm, a non-parametric machine learning classifier. Location within or outside former apartheid “homelands” was taken as a proxy for smallholder, and commercial irrigation. Being in a medium rainfall area, the huge irrigation potential of the Inkomati-Usuthu Water Management Area (UWMA) is already well developed for commercial crop production outside former homelands. However, information about the spatial distribution and extent of irrigated areas within former homelands, which is largely informal, was missing. Therefore, we first classified cultivated lands in 2019 and 2020 as a baseline, from where the Normalised Difference Vegetation Index (NDVI) was used to distinguish irrigated from rainfed, focusing on the dry winter period when crops are predominately irrigated. The mapping accuracy of 84.9% improved the efficacy in defining the actual spatial extent of current irrigated areas at both smallholder and commercial spatial scales. The proportion of irrigated areas was high for both commercial (92.5%) and smallholder (96.2%) irrigation. Moreover, smallholder irrigation increased by over 19% between 2019 and 2020, compared to slightly over 7% in the commercial sector. Such information is critical for policy formulation regarding equitable and inclusive water allocation, irrigation expansion, land reform, and food and water security in smallholder farming systems.

## 1 Introduction

The impacts of climate change, such as increasing temperatures, frequency, and intensity of drought, and flooding, coupled with population growth, urbanisation, land degradation, and improper agricultural practices, are compounding the existing food and water insecurity challenges [1,2]. The projected world population growth to over 9 billion people by 2050 [3] will increase the land under irrigation for agriculture to meet the food requirements of the increased population [4,5]. If not well planned, this will result in socio-ecological unsustainability, compounding climate change, and severe consequences on human and environmental health and wellbeing [6,7]. Apart from the socio-economic benefits, increasing the irrigated area can exacerbate the pressure on already dwindling freshwater resources, which is a cause for concern under climate change [8–11]. The warming climate exacerbates the challenge of water and food insecurity, through unpredictability and scarcity, which have already resulted in shifts in agro-ecological zones and, thus, is affecting crop yields [12]. These impacts are bound to worsen without clear knowledge of the spatial extent of irrigated areas, and dynamic shifts in these [7]. This important information is needed to plan and formulate policies and strategies on irrigation expansion, rural economic development, land reform, and agricultural water management [7].

Irrigation is an indispensable climate change adaptation strategy, especially for small-holder farmers who constitute most farmers in developing countries and are the most vulnerable to climate change [11]. Particularly, their reliance on the increasingly highly variable and unpredictable rainfall for agriculture makes the transition to irrigated agriculture more relevant now than ever [13,14]. However, the cross-cutting challenges of irrigation expansion require cross-sectoral and transformative approaches that recognise the interlinkages within and between systems [15,16]. This is based, in part, on that almost 70% of the available freshwater resource withdrawals are already being used for crop production, on only 18% of cultivated areas globally [4]. Therefore, the worsening climate change, the challenges of rural socio-economic development, and the increasing demand for food, whether it be for household or national food security, or as an economic activity for livelihoods or national gross domestic product (GDP), warrant coherent policies and strategies that balance improved water use efficiency with environmental and human outcomes for sustainability [8,12].

Knowledge of the current spatial extent and dynamic changes in irrigated land is important to inform policy and decision making in formulating coherent strategies on water allocation, agricultural water management, regulating land and water use, and directing irrigation infrastructure investment and development [17]. However, this information is scant, compromising the sustained and transformational change needed in the agriculture sector to enhance water and food security, and socio-ecological sustainability [11,18]. Existing databases on irrigated areas are mostly developed at the global scale and, generally, on a coarse spatial resolution, with a spatial mismatch, therefore misrepresenting the actual irrigated areas [6,11,19]. A more localised spatial scale and resolution are preferred for tracking changes in irrigated areas over time [18,19]. This has become even more important with the increasing vulnerability to climate disasters and other risks, such as the COVID-19 pandemic, which impacted agriculture in ways that are still largely unknown [16]. Thus, accurate data on irrigated land, disaggregated between smallholder and large-scale commercial farming, are essential for informing policy and supporting decision makers with strategies that promote and increase the sustainability of the agriculture sector in terms of economic, social, and environmental dimensions. Such localised quantification is more important for the smallholder sub-sector, which is on small and fragmented parcels of land, which is usually difficult to detect on low resolution satellite images [11,20], and may, therefore, be substantially under-estimated. Because of the mismatch in the scale of regional and global irrigation mapping, the use of irrigation water in smallholder farming areas is largely unaccounted for. Yet, it is widely recognised that the total area of smallholder irrigated areas in many African countries is greater than commercial irrigation [21,22]. As a result, it becomes evident that there is more water use in the smallholder irrigation subsector, yet most of this water is unaccounted for [21,23]. In South Africa, where large-scale commercial white farmers own most irrigated areas, the proportion of cultivated areas that are irrigated was similar for small- and large-scale irrigation in one province, Limpopo [23,24].

This study builds on earlier initiatives that also extracted irrigated areas world-wide [25–27]. These projects produced irrigated area maps that were generally coarse in spatial resolution, as they were mostly regional or global initiatives. These include the FAO (Food and Agriculture Organization) database [28], Global Map of Irrigated Areas version 5 (GMIA 5.0) [29], the MIRCA 2000 product [30], and IWMI’s (International Water Management Institute) irrigated area map [31], among other datasets. In 2016, IWMI developed an improved irrigated area geospatial product for Asia and Africa using Fourier series, canonical correlation analysis, and time-lagged regression at a spatial resolution of 250m for 2000 and 2010 [27]. Subsequently, IWMI also developed another localised irrigated areas map for Limpopo Province in South Africa, using Landsat 8 imagery [11]. Even though these spatial datasets are crucial in quantifying and mapping irrigated areas, the accuracy is generally low due to low spatial resolution [19]. A recent study in South Africa revealed that using low spatial resolution data can lead to the misclassification of irrigated areas, particularly in smallholder fields that are 1 to 2 ha in size, which is too small to be distinguished by low-resolution satellites [19,32].

Advances in information technologies have significantly improved remote sensing tools through the advent of cloud-based big data management platforms such as Google Earth Engine (GEE), artificial intelligence, and machine learning algorithms [6,33]. These advances, coupled with the availability of freely accessible remotely sensed datasets, greatly improve agricultural information management by reducing the pre-processing, processing, and post-processing time [6,19,33–37]. The mapping improvements are concurrently applied to improve the mapping accuracy and distinguish irrigated from rainfed areas [6].

We, therefore, used this improved technology to accurately assess irrigation by small-holder farmers relative to larger-scale commercial irrigation, and how this compares under different extrinsic circumstances. This study developed a more accurate irrigated area dataset for the Inkomati-Usuthu Water Management Area (IUWMA), South Africa, using a combination of the random forest classifier, GEE, and the R-programming language. The IUWMA is appropriate for this study as it is a relatively moist area in an arid country. It has a substantial combination of commercial and smallholder agricultural land use that constitutes a major regional gross domestic product (GDP) component. The water management area has a large, resource-poor, rural population within its former homelands. However, it has substantial high-priority biodiversity conservation areas, including priority mountain catchment areas and important catchments in the world-renowned Kruger National Park. Moreover, there are downstream obligations of water flow and water quality to Mozambique (Sabi and Komati) and Swaziland (Usuthu Rivers); however, all the available freshwater resources are almost all allocated [38]. We then used this increased understanding to inform policy and guide decision making on informed strategies on sustainable irrigation expansion for commercial and smallholder sectors and accounting for the possible impacts of the stochastic events, such as those represented by the COVID-19 pandemic, on agriculture and agricultural water use.

## 2 Methods

### 2.1 Description of the Study Area

**Figure 1:**
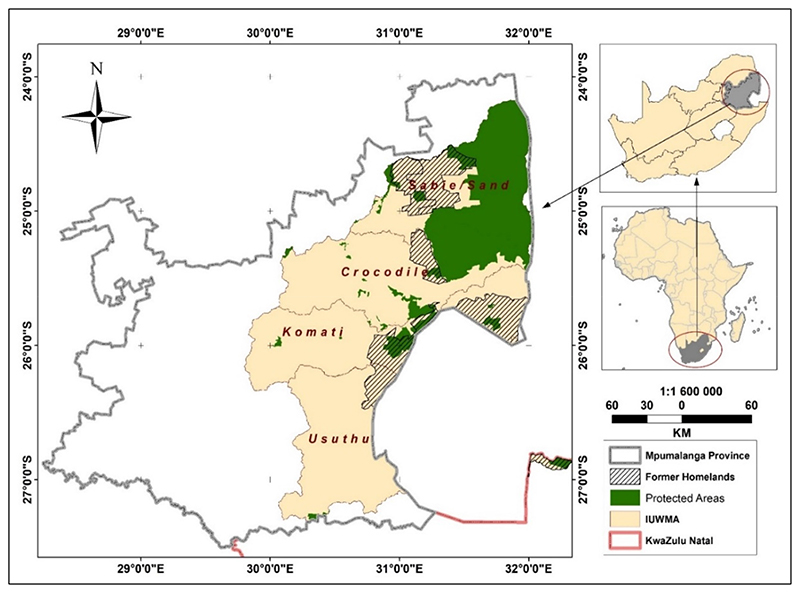
The IUWMA and the sub-catchments also showing former homelands, areas established under apartheid, and reflect the current rural informal smallholder agricultural sector.

The study focused on the 37,000 km^2^ IUWMA (Figure 1), in the eastern half of Mpumalanga Province, with a small component in northern Kwazulu-Natal, and comprises four sub-catchments, including the Sabie/Sand, Usuthu, Crocodile, and Komati Rivers. The IUWMA was established for efficient water management at decentralised levels.

The topography is characterised by a Great Escarpment, dividing its land area into two major sections: (a) the Plateau area with an elevation of more than 2000 m to the west, and (b) the Lowveld area in the east [39]. The two topographic zones determine the climate of the water management area where a temperate climate dominates the Highveld, whilst a sub-tropical climate dominates the Lowveld areas [38]. Rainfall is seasonal, occurring during the summer season (October to February). The IUWMA is a relatively moist region compared to the rest of the country, yet almost all its freshwater resources are allocated, leaving little room for further development [38]. The winter season (April to August) is generally dry and cold, with occasional light snow in the southwestern divide. These relatively warm and almost frost-free conditions make the water management area a vibrant irrigated crop production area during the dry winter season. The average annual temperature is about 20 °C, and the mean annual rainfall ranges from 400 mm to 1500 mm [39]. Natural vegetation is predominantly grassland in the higher altitudes, and savanna at lower altitudes and winter agriculture is irrigated due to the dry conditions. Smallholder irrigation is predominant in former homelands, also called Bantustans (Figure 1), areas allocated to indigenous black people during the apartheid era, which were, and still are, generally poorly resourced. The north-eastern section of the IUWMA lies within the Kruger National Park [40]. The water management area is rich in minerals that include huge coal reserves. However, coal mining seriously affects water quality [39].

### 2.2 Methodological Framework

**Figure 2:**
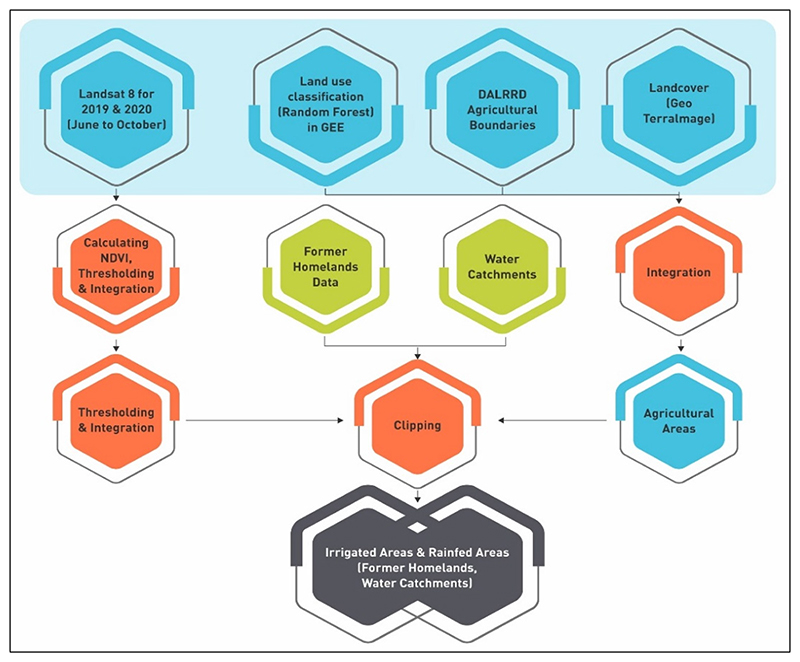
Methodological framework to classify irrigated areas using a non-parametric machine learning algorithm, the random forest.

The methodological framework (Figure 2) illustrates the processes in classifying irrigated and rainfed areas in the IUWMA. High-resolution and cloud-free satellite images from the Sentinel 2 (20 m spatial resolution) for June to October 2020 (a period when irrigation within the tropical region is detectable using the Normalised Difference Vegetation Index (NDVI) as it is dry) were selected and mosaicked within the GEE platform. The mapping exercise facilitated deriving disaggregated statistics for smallholder and commercial farming areas to inform policy formulation and decision making.

The former homelands dataset extracted cultivated areas in predominantly small-holder farming areas, which are generally small and fragmented. Large commercial farming is absent in former homelands. The extraction of smallholder farmlands facilitated distinguishing smallholder croplands from commercial farms in each catchment and deriving related statistics. The GEE functionalities were used to identify four land use categories using Sentinel 2 images (Table 1) through spectral signatures of the four land uses: water, natural vegetation, agricultural areas, and built-up areas.

The focus was on seasonal crops (maize, wheat, soybean, groundnuts, etc.) and we excluded planted permanent fruit crops (orange, banana, macadamia plantations, etc.). Planted fruit crops are differentiated from natural forests in that natural forests in tropical regions shed leaves during the dry winter season, whereas planted fruit crops are always green. Moreover, in almost all cases, planted fruit crop fields maintain a defined shape, and the trees are grown in lines, which facilitates their differentiation. The random forest algorithm can identify and map such land uses to keep track of these attributes and decision rules during the mapping process [41,42].

A supervised classification was run using the random forest classifier. The random forest classifier was chosen mainly because of its flexibility and ability to classify data with a high degree of accuracy [43,44]. The agricultural areas from the classified product were then integrated with already existing agricultural datasets. These datasets include the digitised farm boundaries, which were acquired from the Department of Agriculture, Land Reform, and Rural Development’s (DALRRD) [45], and the 2018 land use map of South Africa, which classifies cropland [46].

### 2.3 Average Monthly Rainfall in IUWMA

**Figure 3:**
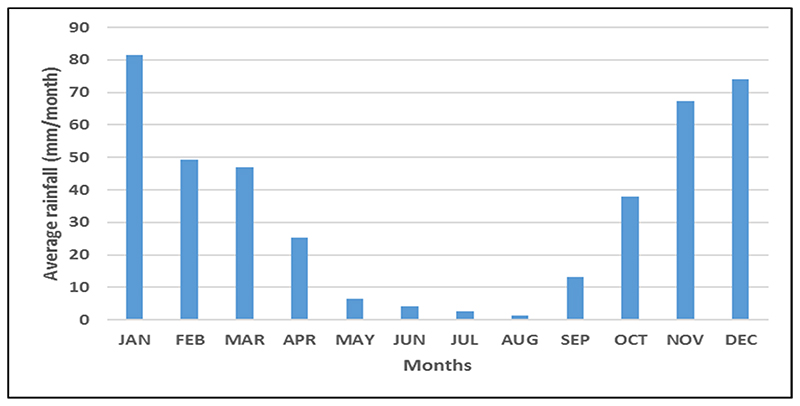
Mean monthly average rainfall in the IUWMA (1972–2020).

The classification of irrigated areas targeted the dry winter seasons (April to August) when little or no rainfall is received in the water management area (Figure 3). Crop production during the dry winter season is, therefore, through irrigation [6,11,47]. Previous studies have indicated that there is crop production throughout the year, but it is divided into two types, irrigated and rainfed agriculture [11,47]. Horticultural crops that include green peas, butternut, tomatoes, potatoes, and dry beans are generally grown during the dry winter period under irrigation using groundwater [47]. Irrigation during the wet summer period is mainly for cash crops at the commercial level and is generally supplementary irrigation for the intra-seasonal dry spells [6,11,47].

### 2.4 Extracting Crop Phenology from NDVI

**Figure 4:**
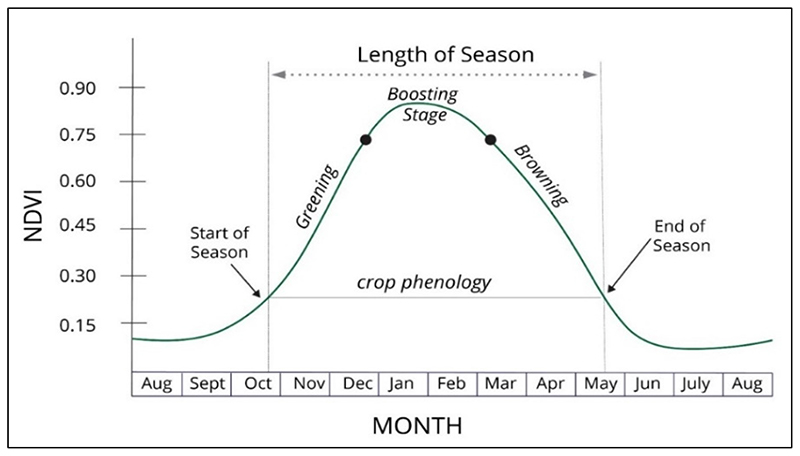
An example of single growing season and related phenological measures analysed through NDVI.

Healthy crops reflect infra-red and absorb red and blue, whereas unhealthy plants reflect red and absorb infra-red portions of the electromagnetic spectrum. So the red and near infra-red bands are important in compiling the NDVI and other vegetation indices, as the blue portion is absorbed in the upper atmosphere [48]. In a region with a single modal annual rainfall pattern, there is less absorption of the visible light and low reflection of the infrared light during the dry season (Figure 4), resulting in low NDVI values. High NDVI values on croplands during the dry season signify irrigation, as there is generally insufficient rainfall to stimulate leaf flush. NDVI values drop around May to June but pick up between July and September due to irrigation (Figure 4).

Outliers and seasonality typically characterise a time series of NDVI data in regions characterised by dry and wet seasons [49]. The single modal annual rainfall pattern of the study area shows that rainfall (or agricultural season) starts in October and ends in April, indicating a time lag between rainfall and seasonal crop phenophases [50] (Figure 3). As rainfall is a key factor in the seasonal crop cycle, there is a positive correlation between rainfall and the seasonal crop growth pattern [51]. Thus, vegetation growth and greening begin in October/November, and browning begins in May/June (Figure 4). This process also applies to rainfed crops in areas with a single modal crop growth cycle. In winter irrigated areas, greening starts at any time of the dry period from June to October. Croplands could be at a greening stage, yet others are at the boosting stage, while some are at the browning stage.

In most cases, farming relies on rainfall during the wet season; the rainfed crop will start greening in November and browning in May. The greening of irrigated crops begins around June and the browning in October. Thus, the classification of irrigated areas was assessed from June to October, coinciding with the dry season.

NDVI time-series data were crucial in the extraction of crop phenology. To extract crop phenology, average monthly NDVI data derived from the Landsat 8 images were computed on GEE from June to October, the winter and dry season for the 2019 and 2020 datasets. The NDVI was used to distinguish irrigated areas from rainfed areas and compare changes in irrigated areas during the 2019 and 2020 winter growing seasons.

### 2.5 Mapping Irrigated and Rainfed Areas

To separate irrigated and non-irrigated areas, NDVI thresholds between 0.19 and 0.25 were employed, and this was in full agreement with the literature [47,52]. These NDVI thresholds were determined using histogram equalisation and the sigmoid contrast stretch [6]. Cell statistics were used to integrate the irrigated and non-irrigated datasets from each year into one dataset. Therefore, the seasonal variations of vegetation indices provided the basis for distinguishing irrigated from rainfed areas using time-series NDVI data. The final dataset was then masked to the extent of the agricultural areas (derived from the integration of three datasets (GeoTerraImage, DALRRD, and the classification performed in this study)).

Agricultural areas were classified using Sentinel 2 imagery, and integrated data from the 2018 South Africa Land cover map. The classified agricultural areas were verified for accuracy assessment using Google Earth imagery. Then the Landsat 8 derived NDVI was used to identify cropped fields during the dry winter season. The kappa index and confusion matrix were used to assess the land use/cover classification accuracy. The ground-truth points used were randomly chosen, and information on each was compared between the land use/cover and Google Earth imagery. The kappa confusion matrix gave an overall accuracy of 84.9%, which is a highly acceptable accuracy. The accuracy assessment is indispensable for determining the quality of the classified cultivated area derived from the random forest classifier.

## 3 Results

### 3.1 Delineating the Irrigated and Rainfed Areas

**Figure 5:**
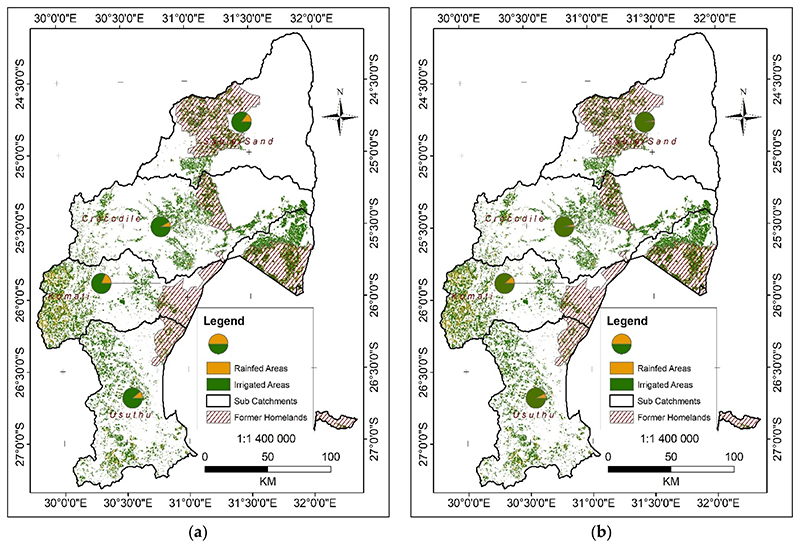
Distribution of cropped areas (irrigated and rainfed) in the IUWMA and the proportion between irrigated and rainfed areas per sub-catchment comparing 2019 (map (**a**)) and 2020 (map (**b**)).

The initial product developed from the remote sensing processing of Sentinel was a map showing the extent and spatial distribution of cultivated areas in both 2019 and 2020 (Figure 5a,b, respectively). The maps also show the proportion of the two cultivation systems per sub-catchment, comparing 2019 and 2020. The cultivated cropland map incorporates both irrigated and rainfed areas. Irrigation outside former homelands is assumed to be large-scale and formal, and within former homelands, irrigation is assumed to be undertaken by smallholders.

The IUWMA has a large area of cultivated cropland (Figure 5), occupying 15% of the total land area. However, the distribution of cultivated land is uneven, as determined by topography, distribution of the river network and soil types, and the location of former homelands (Figure 5). The highest concentration of cultivated land is mainly in the Lowveld area to the east of Komati and Usuthu sub-catchments and the former homelands. The parts of the Sabie and Crocodile sub-catchments bare of agriculture are mostly within the Kruger National Park and other conservation areas.

#### 3.1.1 Changes in Cropped Areas between 2019 and 2020 in Sub-Catchments

The statistical information is given in Tables 2 and 3. It shows the irrigated and rainfed areas in the IUWMA. At over 80% of the total cultivated area, irrigated land is predominant in the water management area, where the rainfed area accounts for less than 10% of the total cultivated area.

#### 3.1.2 Changes in the Cultivated Area between 2019 and 2020 in Former Homelands

**Figure 6:**
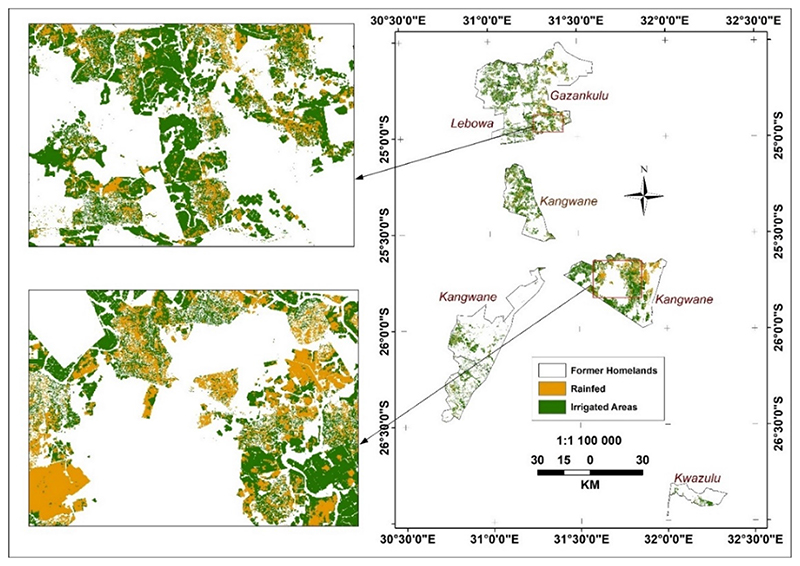
Irrigated and rainfed areas in former homelands in winter 2019.

**Figure 7:**
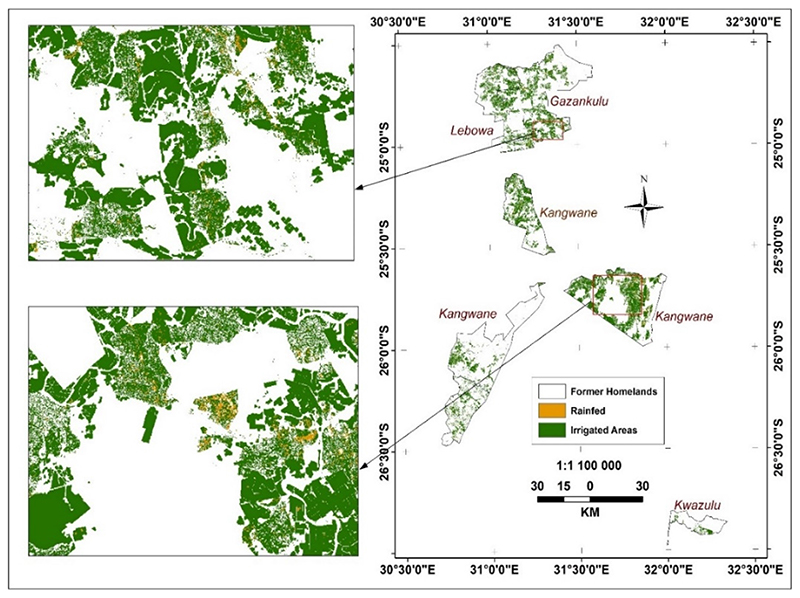
Irrigated and rainfed areas in former homelands in winter 2020.

The same trend of irrigated land predominance continues in former homelands (Figures 6 and 7). Although slightly less in 2019, the land under irrigation increased even more strongly between 2019 and 2020 in the former homelands (Figures 6 and 7 and Tables 4 and 5): from 80% of the total cultivated areas in 2019 to 96% in 2020.

### 3.2 Changes in the Irrigated Area between 2019 and 2020

**Figure 8:**
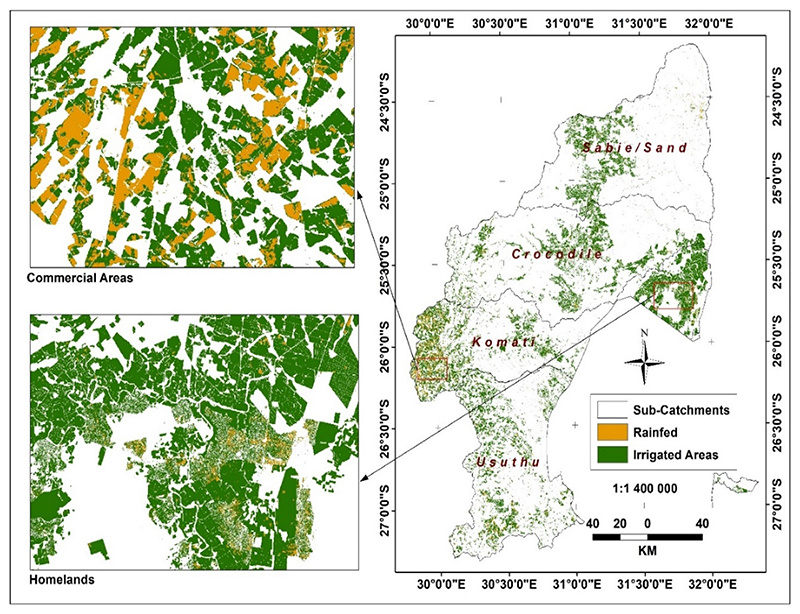
Irrigated and rainfed areas in the IUWMA in winter 2020.

**Figure 9:**
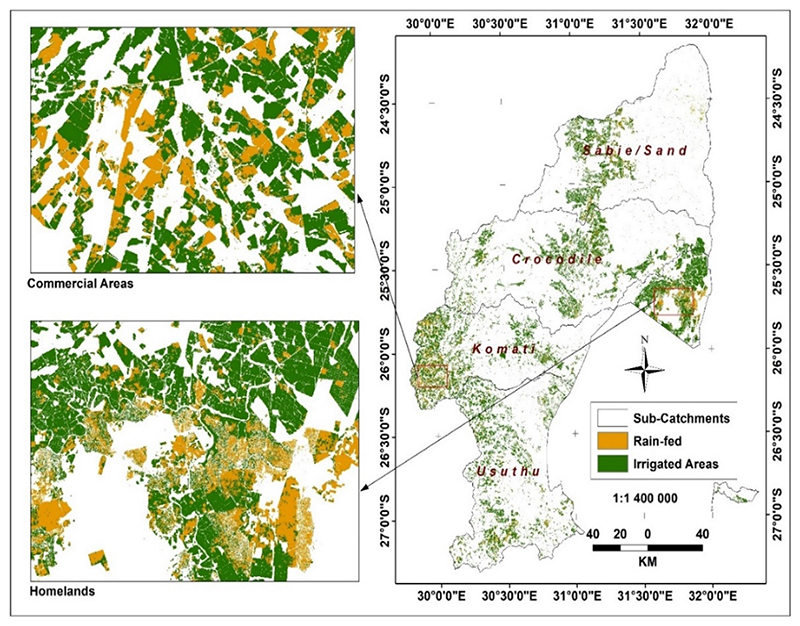
Irrigated and rainfed areas in the IUWMA in winter 2019.

As shown in Tables 2 and 4, with changes summarised in Tables 3 and 5, significant increases are notable in irrigated areas in the IUWMA between the 2019 and 2020 winter growing seasons. This characteristic is evident in all sub-catchments. Figures 8 and 9 further detail how some cultivated areas that were not irrigated in 2019 were irrigated in 2020. Therefore, the increase is not necessarily a change in the area of land under cultivation, but variations in both rainfed and irrigated areas between 2019 and 2020. Although the rainfed area is less than irrigated in the IUWMA, it is also evident that between 2019 and 2020, the area that continued as rainfed further decreased by over 45% (Table 6). During the same period, the land under irrigation increased by 7.3% (Table 6), putting a further strain on already scarce water resources, as highlighted by previous studies [38]. An even stronger trend also manifests in former homelands, where the land under irrigation increased by 19%, and rainfed agriculture decreased by 80% (Table 6).

## 4 Discussion

Irrigated agriculture is fundamental to food and water security as it accounts for 40% of global food production on less than a third of the world’s cropped land [53]. The proportion could increase if the uncounted water used in smallholder irrigation is included [54]. Irrigation is projected to play a pivotal role in future food production because of climate change and associated variability [4,55,56]. Currently, the sub-sector supports food production in dry seasons, generally using groundwater resources in arid and semi-arid regions like South Africa to produce food, and increasingly supplements production during the dry season [6,11]. The importance of irrigated agriculture is evidenced by its high yields, between 30% and 60% higher than rainfed agriculture [57]. However, as already alluded to, irrigation already accounts for over 70% of total global freshwater withdrawals (both surface and groundwater) [4]. Irrigated agriculture, therefore, plays an important role in food and water security. As the population is projected to more than double by 2050 and more than treble by 2100 [3], irrigated agriculture is expected to increase significantly if agriculture is to meet the increasing food demands from a growing population. Plans to increase the land under irrigation should be informed by accurate information on the present spatial distribution and extent of irrigated agriculture, yet this information is very scant [11].

Accurate and up-to-date spatial information on irrigated areas is necessary to effectively manage the limited water resources and is critical for policy decisions that improve water use efficiency, promote irrigation expansion and inform water reallocation [6,11]. The availability of an accurate irrigated agriculture dataset also facilitates strategic on-farm decisions such as irrigation scheduling and improved water productivity throughout the growing season [58,59]. Such knowledge is critical for informing irrigation expansion and enhancing food and water security. Thus, a high resolution and accurate irrigation dataset is essential for assessing irrigation water requirements, cropping patterns, and evapotranspiration trends in highly irrigated areas in space and time [19]. This information facilitates hydrologic modelling that determines groundwater recharge, assessment of water demands at the field scale, and characterising the spatio-temporal variation of crop yields in irrigated areas [19,60].

As population increases and climate change compound water and food insecurity challenges [61], one strategy that can be adopted to ensure food and water security is increasing the irrigated areas to allow crops to be cultivated outside their optimal climate growing regions and buffering against climate variability [4]. This approach makes irrigation an important climate change adaptation strategy. However, irrigation could negatively affect water resources, resulting in groundwater depletion and diminished surface water supplies, as is the current case in the IUWMA, with huge impacts on downstream water supplies and availability [47]. Moreover, irrigation expansion can damage natural habitats and disturb natural environments, with serious socio-ecological impacts, such as biodiversity loss and associated reduction in ecosystem services [62]. Therefore, irrigation guidelines and policies must integrate water and water use policies to ensure sustainable resource use in the irrigation sector. However, this is possible only when there is accurate spatial information on the distribution and extent of current irrigated areas. This is what this study has done, producing an alternative methodology to accurately map irrigated areas, in support of previous studies [6,11,23,47].

As previous studies have shown a significant trend of the increasing area under irrigation and increased water use in irrigated agriculture [6,11,23,47], there is an urgent need for new strategies to enhance the water productivity and avail water resources to other sectors where water is also needed. New strategies also need to acknowledge early gaps and inequalities that exist and dictate water resource access, allocation, and use due to era-specific policies, for example, the apartheid era in South Africa. For example, in such a setting, new strategies, legislation, and policy need to promote resource decentralization and equity and shift towards policy integration for fairness and feasibility of implementing policies. The National Water Act of South Africa [63], highlights, for example, that inequities within the water sector need to be redressed for the gaps within the irrigation sector (between small-holder and commercial irrigation) to be addressed. This means that, where water resources become too limited, poverty alleviation and food security of historically disadvantaged small-scale informal irrigators need to be prioritized over the disproportionately high volumes used by relatively few formal, labour-extensive large-scale irrigators [63]. There is, therefore, a need for policy and decision makers to formulate strategies that promote access to water by smallholder farmers without compromising water security. However, these policies need to be based on accurate spatial information on current irrigated areas. Currently, most smallholder farmers in southern Africa, including South Africa, lack access to water, a situation compounded by poor and biased policies, incorrect water allocation and poor distribution mechanisms, and a lack of institutional environments to account for socio-economic biases and promote equity [64,65].

The climate-sensitive agriculture sector is a prominent feature for the economies of many countries worldwide. In southern Africa, for example, over 70% of the population relies on this sector for food, income, and employment [12]. Yet, the increasing intensity and frequency of extreme weather events of droughts and heatwaves have become a major cause of low yields, and worsening food and water insecurity [12]. The challenges call for urgent interventions to enhance water-efficient cropping systems, and food and water security innovations and strategies that drive related Sustainable Development Goals (SDGs). Given the intensification of climatic changes, pronounced rainfall variability, and water challenges, the Southern African Development Community (SADC) Regional Indicative Strategic Plan (RISDP) of 2003 and the Comprehensive Africa Agriculture Development Programme (CAADP), and Regional Agricultural Policy (RAP), all highlight a need for the agriculture sector to be prioritised while acknowledging that rainfed agriculture alone is exposed to risk and maybe unsustainable as a food security strategy or to drive economic development [14].

In the IUWMA, irrigated areas were 86% of total cultivated areas in 2019, increasing to 92% in 2020. The change was even more pronounced in smallholder irrigation in former homelands, increasing from 80% to 96%. This further underlines the growing importance of irrigation for food security, particularly among the most vulnerable, high-lighting the significant transformations in smallholder agriculture in the management area. The COVID-19 pandemic probably contributed to this remarkable increase in land area under irrigation as households resorted to agriculture to supplement the lost income (www.gov.za/sites/default/files/gcis_document/202005/43321rg11113gon535_0.pdf (accessed on 10 November 2021)). This is based on the fact that agriculture was considered an essential sector that was allowed to operate during COVID-19 pandemic lockdowns. People who lost jobs and other sources of income during the lockdowns resorted to agriculture as an alternative source of income. This highlights the importance of irrigated agriculture in enhancing food security and improving rural livelihoods. Further empirical research and ground-truthing will enhance the method’s accuracy and improve the understanding of the impacts of COVID-19, weather and climate changes, and other contributing factors. Apart from informing policy formulation and decision making, disaggregated information on irrigated areas’ spatial distribution and extent facilitate estimating consumptive water use of crops in irrigated agriculture [47].

Despite being important in improved crop productivity, irrigated agriculture has large water consumption through high unproductive losses through runoff and evaporation. For example, winter crop production is very important in a water-scarce country like South Africa, but this could be limited to certain areas due to high water stress and frost in some dry regions [4]. Thus, the water management area is key for sustainable agriculture, which can improve productivity, national food security, and improved livelihoods through earnings spread throughout the year, employment creation, and foreign exchange earned from exports. This brings to the fore the concept of the water–energy–food (WEF) nexus, which considers the interlinked sectors in integrated resources management [14]. With South Africa’s unemployment rate worsening, winter agriculture provides an important opportunity to create employment, create extra income for poor households, and improve livelihoods and the resilience of poor communities. Thus, winter agriculture could be a climate change adaptation strategy, particularly in rural areas. The IUWMA is ideal for this, being both humid and frost-free for crop production throughout the year.

Producing food with scarce water resources (water productivity) and limited land resources (land productivity) is a major challenge in dry and semi-arid climates like South Africa [4]. As the cultivation of drought-tolerant crops enhances soil fertility and could mitigate agronomic challenges in the dry winter season, it is also very important to grow locally adapted plant species and underutilised indigenous crops that adapt to harsh local conditions [66]. This creates an opportunity to develop a dry season cropping system that contributes to food security, conserves scarce resources, and ultimately alleviates poverty in poor former homeland areas.

### 4.1 Policy Implications

Southern Africa has a large number of underdeveloped rural farming communities.

Given the worsening water scarcity challenges in the region, South Africa included, current and potential contributions of rainfed and irrigated agriculture need to be quantified, based on projected rainfall totals and the cost and availability of irrigation water supply [4,7]. This information is critical for strategic policy formulations that lead to the adaptation of the agriculture sector to the current challenges and the development of the smallholder irrigation sector, including supplementary water for rainfed agriculture, and the adoption of irrigation technologies [12,67]. The current policies, such as the CAADP and the RAP under the SADC Food, Agriculture and Natural Resources FANR Directorate, highlight potential for irrigation, especially informal irrigation, to intensify smallholder agriculture and provide opportunities for smallholder farmers to increase their production, combat hunger, poverty, and food insecurity, and improve their socio-economic standing and resilience to climatic shocks in the SADC region, which is experiencing rainfall variability challenges [12,67].

Irrigation developments in South Africa occurred before 1950 and after the Tomlinson Commission on socio-economic development, which recommended irrigation schemes (“informal”) for subsistence-based farming activities to combat hunger and household food insecurity in 1955 in rural areas [4,7]. These schemes were inadequate to allow rural people to participate in agriculture for economic performance and benefits for improved livelihoods, while the “formal” irrigated agriculture allowed for this to happen [4,7]. To date, irrigation remains an essential component of sustainable agricultural development. Still, it needs to be developed systemically and holistically, considering its intricate interlinkages with other sectors of energy and water [68]. That is because agriculture as a sector and, more specifically, informal irrigated agriculture is vulnerable to extreme weather changes and is expected to be more vulnerable to future climatic shocks [64]. Differential climate change impacts will be expected on the overall agriculture sector in South Africa, but the severity of such impacts will largely be due to underlying conditions relating to equity and socio-economic standing created by past biases of the apartheid era.

Those likely to experience severe impacts are communities in marginal areas that were previously not accounted for in the past policy development [69]. Therefore, radical transformation and climate change adaptation will be largely linked to new strategies and policies’ abilities to account for existing gaps, injustices, and unique socio-economic statuses in South Africa. Such policies should, in their framework, prioritise resource decentralisation sustainably, ensure policy integration, and create policy environments that speak of inequality and aim to achieve equity and inclusion. They will have to provide solutions and actions that will enable their objectives to achieve equity and address injustices. This would help in the journey to achieving objectives of development strategies within the agriculture sector such as the Strategic Plan for South Africa’s Agriculture, Agricultural Policy in South Africa, and the Black Economic Empowerment Framework for Agriculture (AgriBEE), which speak of equity, inclusion, agricultural support for smallholders, competitiveness, and profitability of smallholdings and diversification of structures of production for improved livelihoods and socio-economic statuses in South Africa [70]. This study has enhanced the implementation of these policies by providing an approach that accurately maps the spatial distribution and extent of irrigated areas, disaggregated between smallholder and commercial farming areas.

### 4.2 Limitations

Recent advances in remote sensing and machine learning algorithms have improved the mapping and monitoring of irrigated lands under various environmental conditions in near real-time [6,71]. Its main advantage is that they offer a synoptic overview of irrigated areas in various spectral regions and with temporal frequencies adequate to assess crop growth, maturity, and harvest [6,47]. Big data platforms like the GEE facilitate the comparison of images over a long period, allowing appreciating changes over time. This is apart from the time and cost-effectiveness of remotely sensed data compared to traditional statistical surveys [72]. Improved irrigated areas improve water allocation to farmers, irrigation performance and intensity assessment, and environmental impact assessment, thereby improving irrigation water use efficiency.

The accuracy could even improve by applying machine learning algorithms on high-resolution images such as IKONOS, WorldView, RapidEye, and QUICKBIRD, but these remain too costly. The 6-day revisit time of the Sentinel 1 platform facilitates a more precise crop assessment within seven days, allowing the detection of irrigation events to allow for informed irrigation scheduling [71]. Moreover, as a radar sensor, Sentinel 1 is not affected by cloud cover or other weather events.

One challenge of using remote sensing in mapping irrigated areas is related to its use in humid areas, as there is considerable overlap in spectral signatures between irrigated and rainfed areas. The vegetation is always green, making it difficult to separate irrigated fields from rainfed plots. However, this is being overcome by using temporal data on crop planting, maturity, and harvest in combination with spectral information [19,73]. The limitation of the specified revisit periods of sensors is being overcome by the use of unmanned aerial vehicles (UAVs), which allow user-defined temporal and spatial resolution [19].

## 5 Conclusions

Irrigated agriculture is a sustainable climate adaptation strategy that is acknowledged by strategic plans and policies such as the CAADP and the RAP in the SADC region and the Agricultural Policy and the Strategic Plan for South Africa’s Agriculture. As a strategy, irrigated agriculture could play a role in poverty alleviation, helping the SADC region’s member states move closer to achieving food security and SDG 2, in particular, creating employment opportunities, and encouraging economic growth in the SADC region. This study has enhanced the implementation of these policies and strategies by developing more accurate and disaggregated spatial information on the distribution and extent of irrigated areas within smallholder farming systems. Accurate knowledge of the spatial distribution and extent of irrigated areas facilitates formulating and implementing strategic and coherent policies for transforming water and agriculture within smallholder farming systems. Recent advances in remote sensing and the application of machine learning algorithms facilitated the mapping accuracy of 84.9 of irrigated areas. Apart from the mapping accuracy, the study has differentiated smallholders from commercial irrigated areas and derived statistics for each sub-sector. Smallholder irrigated area was shown to be increasing at a faster rate than the commercial area. The knowledge represents a significant change in irrigation analysis and expansion as it includes both smallholder and commercial farmers, allowing policy and decision makers not to leave anyone behind. Therefore, while acknowledging the importance of material investment in sustainable irrigation expansion, it is equally important to recognise the key issues smallholder farmers encounter and their interconnectedness. Therefore, any interventions related to smallholder irrigation expansion should be undertaken holistically. The initial step to achieve informed reforms in the agriculture sector is the provision of accurate spatial extent and distribution of cropped lands, particularly irrigated areas. This study has provided an improved process to enhance the accuracy of irrigated areas mapping.

## Figures and Tables

**Table 1:** Land-cover classification types used as training samples to classify irrigated areas.

Land Classification	Description
Cultivated land	Land planted with seasonal crops (maize, wheat, soybeans, etc.), newly opened cropped areas, fallow land, etc. The list excludes permanent evergreen tree plantationc and fruit orchards.
Vegetated areas	Shrublands, woodlands, grasslands, natural or planted foreats
Water	All water bodies, including rivers, wetlands, reservoirs, etc.
Built-up area	All settlements, including industrial areas

**Table 2:** Proportions of irrigated and rainfed areas per sub-catchment in 2019 and 2020.

Sub-Catchment	Sub-Catchment Area (ha)	Rainfed Area (ha)	Irrigated Area (ha)	Cultivated Area (ha)	Rainfed Area as % of Cultivated Areas	Irrigated Areas as% of Cultivated Areas	Cultivated Area as % of Catchment Area
			2019				
Usuthu	809,577.1	13,454.3	108,792.8	122,247.1	11.0	89.0	15.1
Crocodile	1,044,273.3	10,910.8	119,671.6	130,582.4	8.4	91.6	12.5
Sabie	930,109.3	11,422.2	66,179.5	77,601.6	14.7	85.3	8.3
Komati	863,975.9	40,165.6	180,662.1	220,827.7	18.2	81.8	25.6
Total	2,793,383.6	75,952.8	475,306.0	551,258.8	13.8	86.2	19.7
			2020				
Usuthu	809,577.1	8741.9	113,505.2	122,247.1	7.2	92.9	15.1
Crocodile	1,044,273.3	4937.1	125,745.3	130,682.4	3.8	96.2	12.5
Sabie	930,109.3	2044.3	75,557.3	77,601.6	2.6	97.4	8.3
Komati	863,975.9	25,648.0	195,179.7	220,827.7	11.6	88.4	25.6
Total	2,793,383.6	41,371.3	509,987.5	551,358.8	7.5	92.5	19.7

**Table 3:** Percentage change per sub-catchment in agricultural systems between 2019 and 2020.

	Rainfed Area (%)	Irrigated Area (%)
**Usuthu**	–35.0	4.3
**Crocodile**	–54.8	5.1
**Sabie**	–82.1	14.2
**Komati**	–36.1	8.0
**Overall**	–45.5	7.3

**Table 4:** Proportions of irrigated and rainfed areas in former homelands in 2019 and 2020.

Former Homelands Name	Homeland Area (ha)	Rainfed Area (ha)	Irrigated Area (ha)	Cultivated Area (ha)	Rainfed Area as % of the Cultivated Areas	Irrigated Areas as % of Cultivated Area	Cultivated Area as % of Homeland Areas
			2019				
Kangwane	344,255.6	20,072.1	73,931.9	94,004.0	21.4	78.7	27.3
Gazankulu	134,944.8	7553.8	29,070.9	36,624.7	20.6	79.4	27.1
Kwazulu	22,264.6	72.4	2022.4	2094.8	3.5	96.5	9.4
Lebowa	75,202.0	1859.3	19,606.1	21,465.4	8.7	91.3	28.5
Total	576,667.0	29,557.6	124,631.3	154,188.9	19.2	80.8	26.7
			2020				
Kangwane	344,255.6	4141.0	89,863.0	94,004.0	4.4	95.6	27.3
Gazankulu	134,944.8	1185.3	35,439.5	36,624.7	3.2	96.8	27.1
Kwazulu	22,264.6	1.5	2093.2	2094.8	w0.1	99.9	9.4
Lebowa	75,202.0	508.2	20,957.2	21,465.4	2.4	97.6	28.5
Total	576,667.0	5836.1	148,352.8	154,188.9	3.8	96.2	26.7

**Table 5:** Percentage change in agricultural systems in former homelands between 2019 and 2020.

	Rainfed Area (%)	Irrigated Areas (%)
Kangwane	–79.4	21.6
Gazankulu	–84.3	21.9
Kwazulu	–97.9	3.5
Lebowa	–72.7	6.9
Overall	–80.3	19.0

**Table 6:** Percentage change in the cultivated areas between 2019 and 2020.

	Agriculture Type	2019	2020	% Change
IUMMA	Rain fed areas (ha)	75,966.5	41,282.0	–45.7
	Irrigated areas (ha)	475,360.0	510,044.6	7.3
	Total cultivated areas (ha)	551,326.5	551,326.5	
Former Homelands	Rain fed areas (ha)	29,557.6	5836.1	–80.3
	Irrigated areas (ha)	124,631.3	148,352.8	19.0
	Total homeland (ha)	154,188.9	154,188.9	
Formal/commercial irrigation	Rain fed areas (ha)	46,408.9	35,445.9	–23.6
	Irrigated areas (ha)	350,728.7	361,691.8	3.1
	Total cultivated areas (ha)	397,137.6	397,137.6	

Note: Negative changes represent decrease and positive, increase.

## Data Availability

Publicly available datasets were analysed in this study. These data can be found here: (https://developers.google.com/earth-engine/datasets/catalog/ (accessed on 3 March 2021).
